# A novel endoscopic suture anchor device for large mucosal defect closure in an in vivo porcine model

**DOI:** 10.1055/a-2650-9543

**Published:** 2025-07-29

**Authors:** Jiancong Feng, Yaqi Zhai, Zhenyu Liu, Enqiang Linghu

**Affiliations:** 1 Department of Gastroenterology, The First Medical Center of Chinese PLA General Hospital, Beijing, China

Closure of large mucosal defects following endoscopic submucosal dissection is essential in interventional endoscopy 1 . While multiple closure systems have emerged, their clinical implementation remains constrained by technical complexity and high costs 2 3 , highlighting the need for simple and cost-effective closure devices.

Our research team has designed a novel endoscopic suture anchor device, developed from a conventional through-the-scope clip ( Fig. 1 ). It is compatible with 3.2-mm working channels and integrates sutures for defect closure. This study evaluated the feasibility of the suture anchor device for closing large mucosal defects in an in vivo porcine model.

**Fig. 1:**
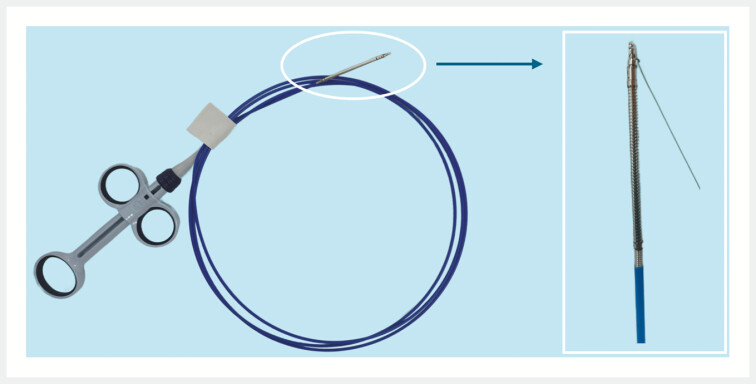
A novel endoscopic suture anchor device (fabricated by Micro-Tech, Nanjing, China).

Defect closure was performed according to the following steps ( Video 1 ). The first suture anchor, pre-secured with a suture, is delivered via the working channel to the mucosal defect margin ( Fig. 2 **a** ). The handle is rotated to drive the anchor into the tissue and then pressed to release the anchor. The second anchor is threaded through the suture and released on the contralateral defect margin via the working channel in the same manner. Tension is applied to the suture to eliminate slack ( Fig. 2 **b** ), then the suture anchors are positioned in a zigzag pattern ( Fig. 2 **c** ). Finally, moderate tension is applied to the suture to approximate the defect edges; the suture is then cut with a suture cinch ( Fig. 2 **d** ). In this case, mucosal closure was achieved with five suture anchors, and no delayed bleeding or perforation was observed at 1-week follow-up ( Fig. 3 ).

The steps for defect closure using the novel endoscopic suture anchors.Video 1

**Fig. 2:**
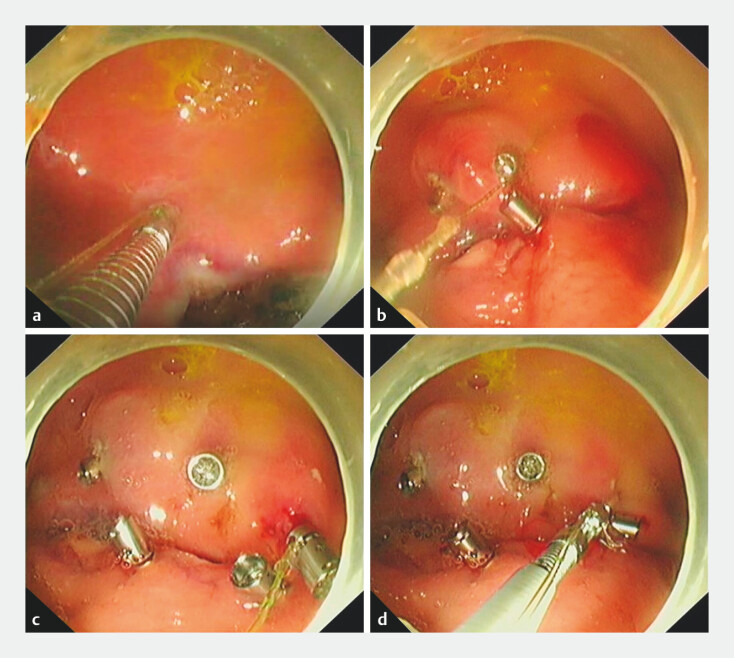
Procedure for closing a mucosal defect using the novel endoscopic suture anchors. **a** The first suture-loaded anchor was deployed 5–10 mm from the defect margin. **b** Tension was applied to the suture to eliminate slack. **c** The suture anchors were positioned in a zigzag pattern. **d** Tension was applied to the suture to approximate the edges; the suture was then cut with a cinch.

**Fig. 3:**
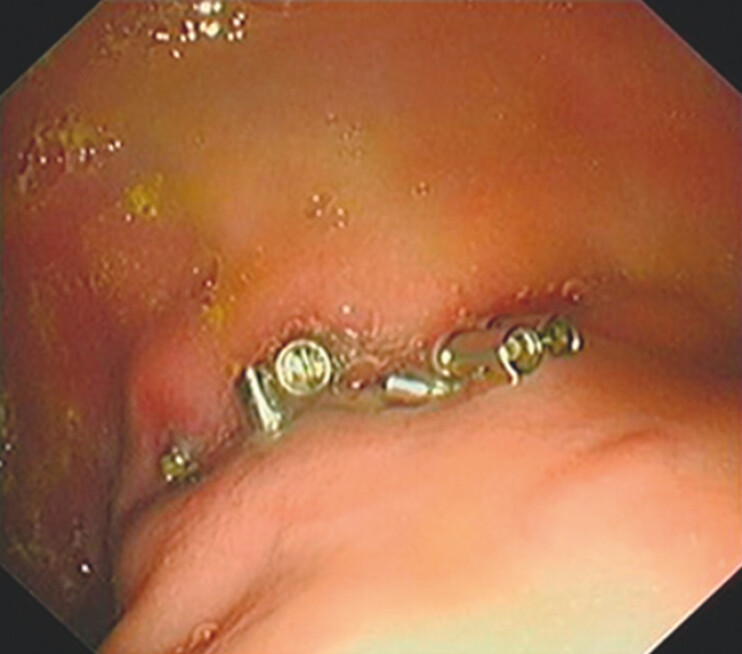
Endoscopic follow-up at 1-week postoperatively revealed no complications.

Suture anchors can be deployed without endoscope withdrawal, and misplaced anchors are retrievable through counter-rotation of the handle. Unlike the X-tack system (Apollo Endosurgery, Inc., Austin, Texas, USA) 4 , suture anchors are delivered through the working channel without requiring a mounting platform, with the single-unit design enabling the quantity to be adjusted to the defect size. The endoscopic suture anchor device demonstrates feasibility for large mucosal defect closure, but further clinical validation is required.

Endoscopy_UCTN_Code_TTT_1AO_2AO

## Notes

Endoscopy E-Videos https://eref.thieme.de/e-videos
*E-Videos* is an open access online section of the journal *Endoscopy* , reporting on interesting cases and new techniques in gastroenterological endoscopy. All papers include a high-quality video and are published with a Creative Commons CC-BY license. Endoscopy E-Videos qualify for HINARI discounts and waivers and eligibility is automatically checked during the submission process. We grant 100% waivers to articles whose corresponding authors are based in Group A countries and 50% waivers to those who are based in Group B countries as classified by Research4Life (see: https://www.research4life.org/access/eligibility/ ). This section has its own submission website at https://mc.manuscriptcentral.com/e-videos .
